# Role of mesenchymal stem cells in meniscal repair

**DOI:** 10.1186/s40634-014-0012-y

**Published:** 2014-09-02

**Authors:** Peter Angele, Richard Kujat, Matthias Koch, Johannes Zellner

**Affiliations:** University Medical Center Regensburg, Franz-Josef-Strauss-Allee 11, 93053 Regensburg, Germany; Sporthopaedicum Regensburg/Straubing, Hildegard von Bingen Strasse 1, 93053 Regensburg, Germany

**Keywords:** Mesenchymal stem cells, Meniscus tear, Meniscus suture, Meniscus transplantation, Biomaterial

## Abstract

Meniscus integrity is the key for joint health of the knee. Therefore, the main goal of every meniscus treatment should be the maintenance of as much meniscus tissue as possible.

Repair of meniscus tears can be achieved by meniscus suture. However, in a recently published meta-analysis, the long-term outcome of meniscus repair showed a mean failure rate of 24%.

In a preclinical trial, locally applied mesenchymal stem cells produced differentiated meniscus-like tissue in meniscus tears indicating that mesenchymal-based cells, harvested from the bone marrow, enhance meniscus healing in critical-size meniscus tears.

Symptomatic meniscus defects offer the option for meniscus transplantation with porous cell free biomaterials, when a complete meniscus rim is available. Cell-free biomaterials, which are actually in clinical application, reveal variable outcome in mid-term results from complete failure to regeneration with meniscus-like tissue.

In several preclinical studies with different critical-size defects in the meniscus, the application of mesenchymal stem cells could significantly enhance meniscus regeneration compared to empty defects or to cell-free biomaterials.

Regenerative treatment of meniscus with mesenchymal stem cells seems to be a promising approach to treat meniscal tears and defects. However it is still not clear, whether the stem cell effect is a direct action of the mesenchymal-based cells or is rather mediated by secretion of certain stimulating factors. The missing knowledge of the underlying mechanism is one of the reasons for regulatory burdens to permit these stem cell-based strategies in clinical practice. Other limitations are the necessity to expand cells prior to transplantation resulting in high treatment costs. Alternative treatment modalities, which use growth factors concentrated from peripheral blood aspirates or mononucleated cells concentrated from bone marrow aspirates, are currently in development in order to allow an attractive one-step procedure without the need for cell expansion in cultures and thus lower efforts and costs.

In summary, Tissue Engineering of meniscus with mesenchymal based cells seems to be a promising approach to treat meniscal tears and defects in order to restore native meniscus tissue. However, advances of the technology are necessary to allow clinical application of this modern regenerative therapy.

## Introduction

Meniscal lesions represent the most common intra-articular knee injury, and are the most frequent cause of surgical procedures performed by orthopaedic surgeons (Makris et al. 2011). The mean annual incidence of meniscal lesions has been reported to be 66 per 100.000 inhabitants, 61 of which result in meniscectomy (Makris et al. 2011). The changes in “pivoting” sports activities in the past few decades have resulted in increased injury rates of the meniscus (1.5 million injuries in Germany per year) (Stein et al. 2010). Especially in combination with anterior cruciate ligament injuries a high incidence of meniscal lesions (40-80%) can be detected.

Meniscus integrity is the key for joint health of the knee. Untreated meniscus tears cause intermittent pain, joint swelling, recurrent mechanical symptoms (clicking, catching, giving way) and, therefore, significant reduction in quality of life in predominately young and active patients (McDermott 2011).

In the long-term, meniscus tears can result in the onset of joint degeneration and, finally, knee osteoarthritis with all its consequences including pain, immobility and knee arthroplasty (Lohmander et al. 2007; Stein et al. 2010; Borchers et al. 2011; Jeong et al. 2012; Badlani et al. 2013). In a recent published case–control study (Level of evidence 3) specific meniscus tear morphologies (Meniscus extrusion, complex tears, tears with large radial involvement) have shown to be significantly more common in patients with progressive development of osteoarthritic changes in a 2 year follow-up indicating that these meniscus tears represent a negative prognostic risk factor for later development of osteoarthritis (Badlani et al. 2013).

Removal of meniscus tears lead to short term relief of clinical symptoms, but to knee osteoarthitis in long-term (Salata et al. 2010; Paxton et al. 2011; Papalia et al. 2011; Jeong et al. 2012). Especially the amount meniscus removed, lateral meniscectomy and longer duration of clinical symptoms preoperatively have been identified as negative prognostic risk factors for the onset of osteoarthritis in systematic reviews (Papalia et al. 2011; Jeong et al. 2012). Elevated expression levels of arthritis-related markers in meniscus tears in patients under forty years old, compared to patients over forty years, and in patients with meniscus and anterior cruciate ligament tears, compared to patients with isolated meniscus tears, indicate an increased catabolic response suggesting a higher risk for progression of osteoarthritis following partial meniscectomy (Brophy and Matava 2012).

Knowing the risk for the onset of osteoarthritis after meniscectomy, the majority of meniscus tears are still treated with partial meniscectomy as shown in a huge cohort of more than 1000 young patients undergoing anterior cruciate ligament reconstruction (Fetzer et al. 2009).

Therefore, the main goal of every meniscus treatment should be the maintenance of as much meniscus tissue as possible (Fetzer et al. 2009; Starke et al. 2009; Stein et al. 2010; Abrams et al. 2013). This includes repair of meniscus tears and regeneration of meniscus defects after meniscectomy with regenerative treatment approaches.

In recent years, there has been a growing interest in using mesenchymal stem cells to regenerate damaged tissue such as injured hyaline cartilage or meniscus. These mesenchymal stem cells fulfill a dual role for musculoskeletal repair, because they have the potential to differentiate into the repair cells themselves and to produce special growth factors for its repair (Caplan and Dennis 2006). This review focuses on the present and future treatment options especially for meniscus repair and meniscus regeneration given by the use of intrinsic or exogenous applied mesenchymal stem cells.

## Review

### Endogenous repair cells of meniscus injury

Currently, a number of potential repair cells for meniscus regeneration are available. Repair cells of meniscus injury can either be located in the meniscus tissue itself or entering the meniscus predominately via circulation. In the following section, we will focus on the availability of repair cells and repair potential of the meniscus.

Endogenous repair of meniscus injury seems to be dependent of the different vascularisation of the outer and the inner zone of the meniscus (Arnoczky 1999). Repair in the vascularized outer zone can be achieved (McDermott 2011), but fail to encourage healing in the avascular inner zone of the meniscus (Hasan et al. 2014; Makris et al. 2011). However, in several studies also regeneration could be seen in the inner zone of the meniscus indicating regenerative potential independently from the vascularization (Nakata et al. 2001; Hennerbichler et al. 2007; Baker et al. 2009; Pabbruwe et al. 2010). Hennerbichler et al. have shown in an experimental setup, that punch defects, which were directly filled with the removed punches, showed no significant difference in healing potential between the vascularized and avascularized meniscus zone (Hennerbichler et al. 2007). Croutze and coworkers could demonstrate equivalent differentiation potential toward chondrogenic phenotype and extracellular matrix production of isolated human meniscus cells from the inner and the outer zone (Croutze et al. 2013). Different studies stated that milieu factors like oxygen tension (Croutze et al. 2013) and growth factors (Pabbruwe et al. 2010) may play a key role in modulating redifferentiation of meniscal fibrochondrocytes at least *in vitro*.

From a clinical standpoint, meniscus cells from meniscectomised tissue would be the ideal cell source for repair. Is this approach applicable? Nakata et al. have shown feasibility of meniscus regeneration by expansion of human meniscus cells from meniscectomised tissue (Nakata et al. 2001). With the same cell source Baker et al. could achieve repair tissue with equivalent mechanical properties than native meniscus tissue (Baker et al. 2009). However the low proliferation rate and matrix production limit the use of this cell type (Nakata et al. 2001). In addition, potential candidates for meniscus transplantation have already undergone partial meniscectomy with the necessity to find alternative cell sources.

Stem cells are characterized by self-renewal capacity and multilineage differentiation potential to a variety of cell types of mesenchymal tissue like bone, cartilage or fat (Caplan 1991; Caplan and Dennis 2006). Recently, the stem cell perspective has changed by identification of pericytes around almost every blood vessel in the body, which contain stem cell characteristics (Bianco et al. 2008; Bianco 2011; Crisan et al. 2008; Crisan et al. 2011). The existing traditional view, which focuses on the multipotent differentiation capacity of these cells, has been expanded to include their equally interesting role as cellular modulators that bring them into a broader therapeutic scenario (Caplan and Correa 2011).

With this background, it is not surprising that Osawa et al. have identified in the vascularized region of the meniscus more blood-vessel derived stem cells (CD34- and CD146-positive cells) than in the avascular region. These meniscal-derived stem cells were multipotent and contributed to meniscal regeneration, which they proof via transplantation in knee joints of athymic rats (Osawa et al. 2013).

Does also the avascular inner zone of the meniscus contain stem cells? Stem cells have been identified in the surface zone of other avascular tissue, mainly in the articular cartilage (Archer et al. 1994; Dowthwaite et al. 2004; McCarthy et al. 2012), but little is known about meniscus stem cells. Mauck et al. describes regional multilineage differentiation potential of meniscus cells in both zones available, however showed differences in pluripotency between the zones (Mauck et al. 2007). Especially, pluripotent cells from the avascular tissue seem to lack osteogenic differentiation potential, which could be of clinical interest for meniscus regenerative approaches (Mauck et al. 2007; McCarthy et al. 2012).

Besides existence of mesenchymal stem cells in the meniscus, the synovium and the synovial fluid contain stem cells for meniscus regeneration (Sakaguchi et al. 2005; Horie et al. 2009; Matsukura et al. 2014). Matsukura and coworkers found elevated levels of mesenchymal stem cells in the synovium fluid after meniscus injury compared to normal knee joints suggesting that mesenchymal stem cells in the fluid may play a role in regeneration of meniscus (Matsukura et al. 2014). Horie and coworkers showed promotion of meniscal regeneration in rat massive meniscal defects by intra-articular injection of synovial stem cells. The injected cells adhere to the lesion site, directly differentiate in meniscus cells and promote meniscal regeneration without mobilization to distant organs (Horie et al. 2009).

In summary, local or systemic stem cells seem to play a fundamental and essential role in the regeneration of meniscus injury, either as direct repair cells or as a source for secretion of bioactive modulators.

### Repair of meniscus tears with meniscus suture

If the meniscus repair succeeds then the patient is left with a normal or near-normal meniscus, and thus no increased risk of osteoarthritis in the future (McDermott 2011; Stein et al. 2010; Paxton et al. 2011). In a cohort study (Level of evidence 3) no osteoarthritic progress was detectable in 80% of the patients after meniscus repair compared with 40% after meniscectomy in a long-term follow-up (mean 9 years) (Stein et al. 2010). In a systematic review meniscal repair showed superior clinical outcome detected by higher Lysholm scores and less radiologic degeneration than meniscectomy (Paxton et al. 2011). However, in a recently published meta-analysis, the long-term outcome of meniscal repair showed a mean failure rate of 23,1% (Nepple et al. 2012).

### Augmentation of meniscus suture

Given the low healing potential of meniscus in the avascular zone and the moderate healing rates of meniscus repair in the vascular zone, there is a strong need for true regeneration of viable and biomechanically functional meniscus tissue.

**Figure 1:**
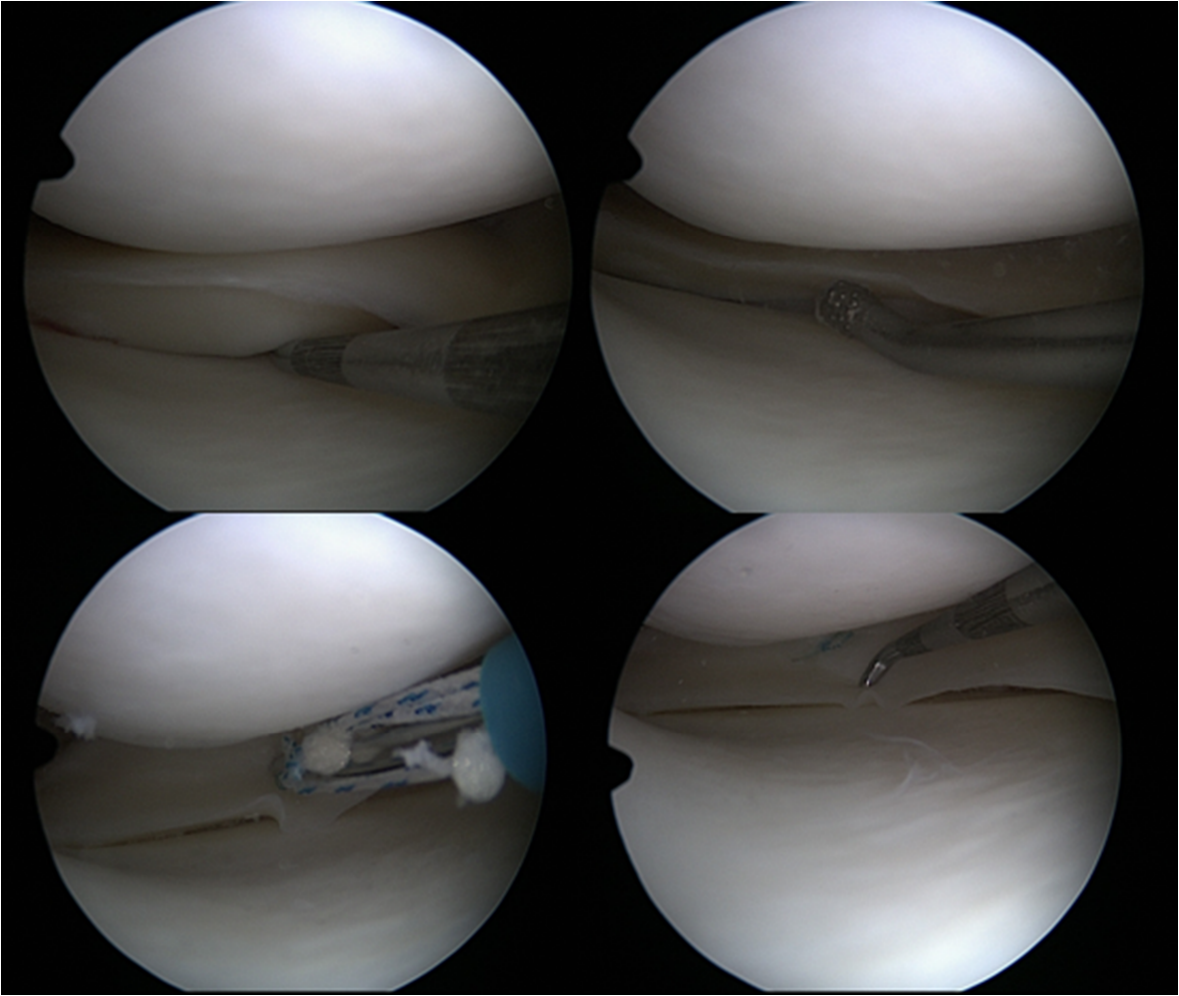
**Medial meniscus tear and meniscus repair in a right knee joint: Meniscus tear recognized by a probe (upper left); Meniscus tear preparation with a grasp, which refreshes the tear site (upper right); Meniscus suture with all inside technique (Fast T Fix, Smith nephew) (lower left); Stable meniscus tear after repair (lower right).**

Several methods have been devised to aid the healing of the meniscus, ranging from refreshing the meniscus tears with rasps and creation of access channels in the meniscus base to introduce blood and reparative cells to meniscus tears (Scotti et al. 2013) (Figure 1). The use of fibrin clots, synovial flaps and resorbable scaffolds in or around the tears were also described in the literature in order to enhance meniscus healing potential (Hasan et al. 2013; Starke et al. 2009).

Exogenous fibrin clots seem to improve healing in animal models and in humans (Starke et al. 2009). It is postulated that the clot serves as a chemotactic and mitogenous stimulus. Platelet-rich plasma (PRP), which contains the majority of growth factors in the peripheral blood, seems to promote the clinical healing of meniscus tears even in the white-white zone (Wei et al. 2012) mainly in the earlier period after meniscal suture. However, the clinical use of this intriguing arthroscopically applicable, one-step procedure for meniscus repair is actually not tested in prospective trials due to regulatory burdens.

### Augmentation of meniscus suture with mesenchymal stem cells

**Figure 2:**
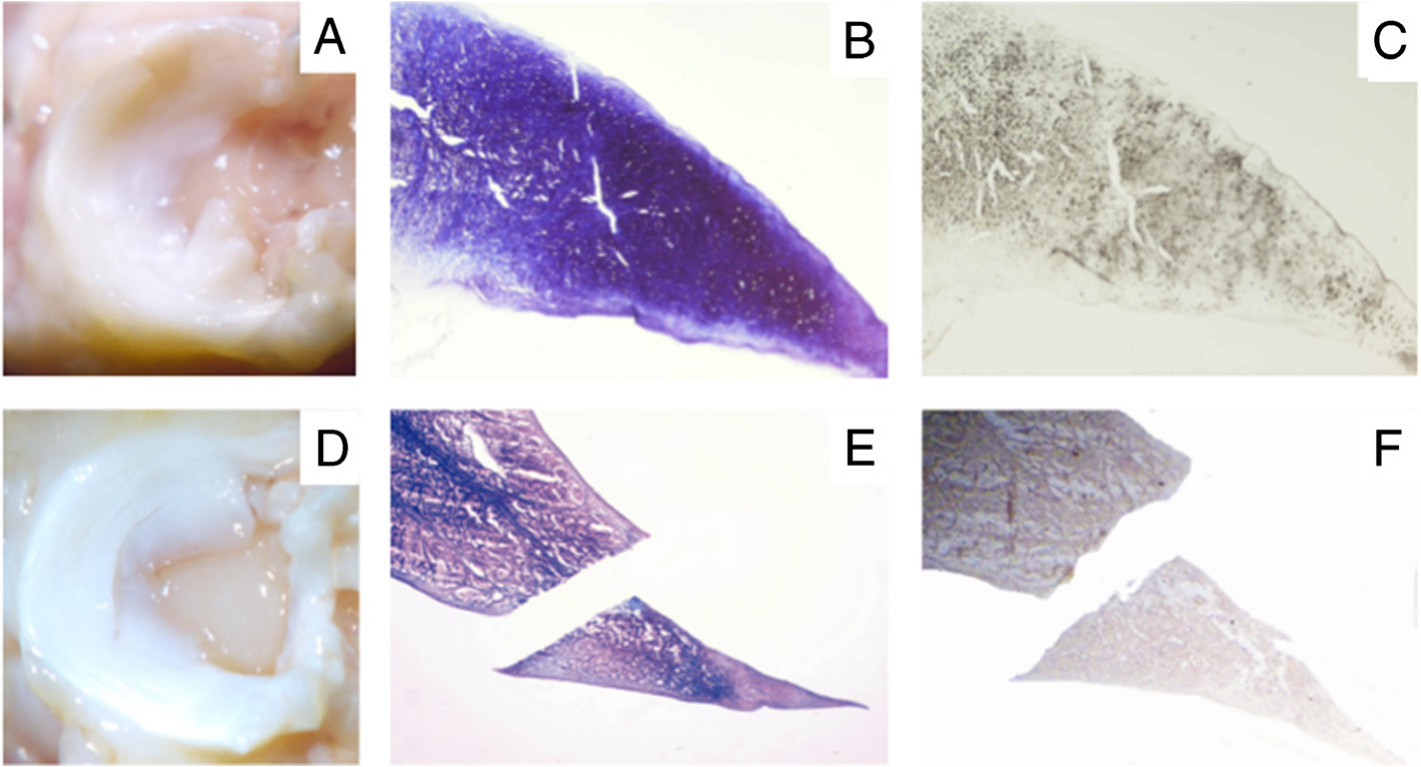
**Treatment of meniscal tears (rabbit) in the avascular zone with a mesenchymal stem cell matrix composite resulted in a stable repair compared to implantation of a cell free scaffold: Representative macroscopic views, histological (toluidine blue) and immunohistochemical (collagen type II) slides of menisci and the meniscal repair tissue 3 months after treatment of tears in the avascular zone: A, B, C: treatment with hyaluronan-collagen composite matrices loaded with mesenchymal stem cells; D, E, F: treatment with a cell-free composite matrix (control).**

Preclinical trials have shown enhanced healing of meniscal lesions with the application of mesenchymal based cells (Table 1) (Table 2) (Hasan et al. 2013; Makris et al. 2011; Izuta et al. 2005; Angele et al. 2008; Zellner et al. 2010; Zellner et al. 2013). Locally applied expanded mesenchymal stem cells from the bone marrow have achieved regeneration of longitudinal meniscus tears in the avascular zone in the lateral meniscus of New Zealand White Rabbits. Control groups with untreated tears, treatment with meniscus suture alone or meniscus suture in combination with implanted cell-free biomaterials revealed no recognizable healing indicating the development of a critical size meniscus tear model. In contrast, mesenchymal stem cells from the bone marrow in combination with hyaluronan collagen carriers resulted in meniscal repair with differentiated meniscus-like tissue detected by histology, immunohistochemistry and biomechanical analysis (Figure 2) (Zellner et al. 2013).

**Table 1:** **Mesenchymal stem cells for meniscus regeneration**

**Pro/advantages**	**Cons/disadvantages**
• High regenerative potential	• High treatment costs
• Self renewal capacity	• Regulatory burden
• Cellular modulation at lesion site	• Missing knowledge of underlying repair mechanisms
• Potential of differentiation into meniscal repair cells	• Possible need for cell expansions prior to application
• Secretion of bioactive substances like growth factors	
• Support/promotion of intrinsic meniscal healing capacity

**Table 2:** **Regenerative treatment options for meniscal lesions**

**Treatment**	**Pro/Cons**
Augmentation of meniscal suture and meniscus reconstruction by intraarticular microfracturing	+ Easy to perform
	+ Adhesion of stem cells at lesion site
	- Uncertain effect
	- Low concentration of stem cells at defect site
Augmentation of meniscal suture or meniscus cell-free reconstruction (e.g. Actifit) by locally applied growth factors (e.g. PRP)	+ Application directly at meniscal lesion site
	+ High concentration at lesion site
	+ Support of intrinsic healing potential
	+ One-step-procedure
	- Preparation time
	- Short term effect at lesion site
	- Uncertain local effect
Augmentation of meniscal suture by locally applied MSCs (e.g. bone marrow derived)	+ High potential differentiation
	+ Application directly at meniscal lesion site
	+ Support of meniscus regeneration
	+ Use of autologous cells
	+ Potentially one-step-procedure
	- Preparation time
	- Regulatory burden
	- Missing knowledge of repair mechanisms
Intraarticular injection of MSCs/growth factors	+ Adhesion at lesion site
	+ Easy to perform
	- Uncertain effect
	- Low concentration of stem cells at lesion site
	- Harvesting and preparation prior to application
	- Side effects in the knee joint beside the defect site
Intravascular injection of MSCs	+ Adhesion at lesion site
	- Uncertain effect
	- Low concentration of stem cells at lesion site
	- Harvesting and preparation prior to application
	- Side effects in other areas besides the defect
	- No clinical experience
Implantation of MSC loaded carrier/scaffold at meniscal defect site	+ Potential for treatment of meniscal critical size defects
	+ Application directly at meniscal defect site
	+ Option for pre-differentiation of MSC/carrier construct
	+ Use of autologous cells
	- High costs
	- Missing knowledge of repair mechanisms
	- Necessity of cell expansion prior to implantation/two-step-procedure
	- No clinical experience

It is not clear whether this is a direct action of the mesenchymal-based cells or is rather mediated by secretion of certain stimulating factors (Starke et al. 2009). Despite the fact, that meniscus regeneration seems to be feasible by growth factors and mononucleated cells, none of the cell-based strategies has entered clinical practice to date (Scotti et al. 2013). The implementation of cell-based strategies is mainly limited by regulatory burdens and by the necessity to expand cells prior to transplantation resulting in high treatment costs.

### Regeneration of meniscus defects

**Figure 3:**
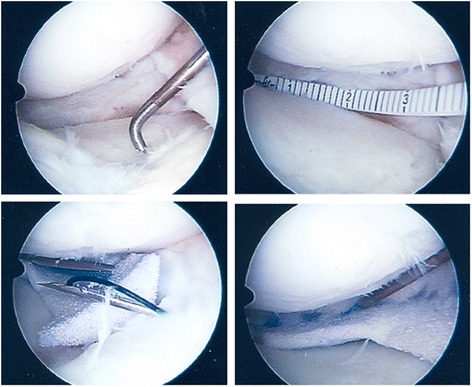
**Meniscus transplantation with cell-free biomaterial after subtotal meniscectomy of the medial meniscus (Actifit).** Subtotal meniscectomy, note the residual meniscus base (upper left); Measurement of the meniscus defect for preparation of suitable biomaterial (upper right); Insertion of the cell-free biomaterial and fixation with all-inside and outside-in suture techniques (lower row).

Searching for optimal restoration of meniscal tissue, biocompatible scaffolds came in the focus for treatment of large meniscal defects in the last decade. The rationale for using cell-free biomaterials after extensive loss of meniscal tissue is based on repopulation of the scaffold by host cells recruited from the synovium or the meniscal remnants (Scotti et al. 2013) (Figure 3).

Two biomaterials are currently in clinical use. The collagen meniscus implant (CMI) was the first scaffold for meniscus replacement. Some authors report satisfactory clinical outcome (Rodkey et al. 1999) while MRI and histological results are controversial. Steadman and Rodkey (2005) saw a tendency of shrinking over time and no histological remnants of the collagen scaffold 5–6 years after implantation (Steadman and Rodkey 2005). Martinek et al. (2006) described predominantly scar tissue formation instead of fibrocartilage after implantation (Martinek et al. 2006). The second biomaterial is a polyurethane based scaffold called Actifit. The first 12 month report showed tissue ingrowth into the scaffold at this time and consistent regeneration in MRI and biopsies (Verdonk et al. 2011).

However, there are no data on the mid- and long term effects of the CMI or the Actifit on the surrounding cartilage in order to prove the beneficial effect on prevention of early onset of osteoarthritis after loss of meniscus tissue. The amount of the developed repair tissue and its quality induced by the cell-free implants still seem to be crucial and improvable. Scar formation, repair tissue rich of vessels and vascularization into the tip of the new meniscus tissue show the need of improvement of this treatment technique. Additionally the biomechanical stability and the intraoperative handling have to be optimized for better clinical results. At the moment the cell-free meniscus implants are only used in a very selective group of patients. Despite promising short term results of meniscal implants, none of them has currently demonstrated regeneration of a functional, long-lasting meniscal tissue.

As biomaterials for meniscal substitution need a stable and intact rim of the native meniscus, allogenic meniscal transplantation is actually the only biologic treatment option available for totally meniscectomized knee joints (Lubowitz et al. 2007). A recently published meta-analysis concluded that this procedure is an effective technique for these very selected patients (Elattar et al. 2011). However fixation of the allograft, the limited availability and the frequent mismatch of graft and host tissue (Shaffer et al. 2000) are still problems of this procedure. In addition, long term protection from joint degeneration has not yet been demonstrated (Wirth et al. 2002).

### Stem cells for regeneration of meniscus defects

In experimental trials different settings have been tested for mesenchymal stem cell based Tissue Engineering approaches for treatment of meniscus defects (Table 1). Stem cells can be administered by different ways of application. In most studies, the local application of mesenchymal stem cells has been used (Table 2).

**Figure 4:**
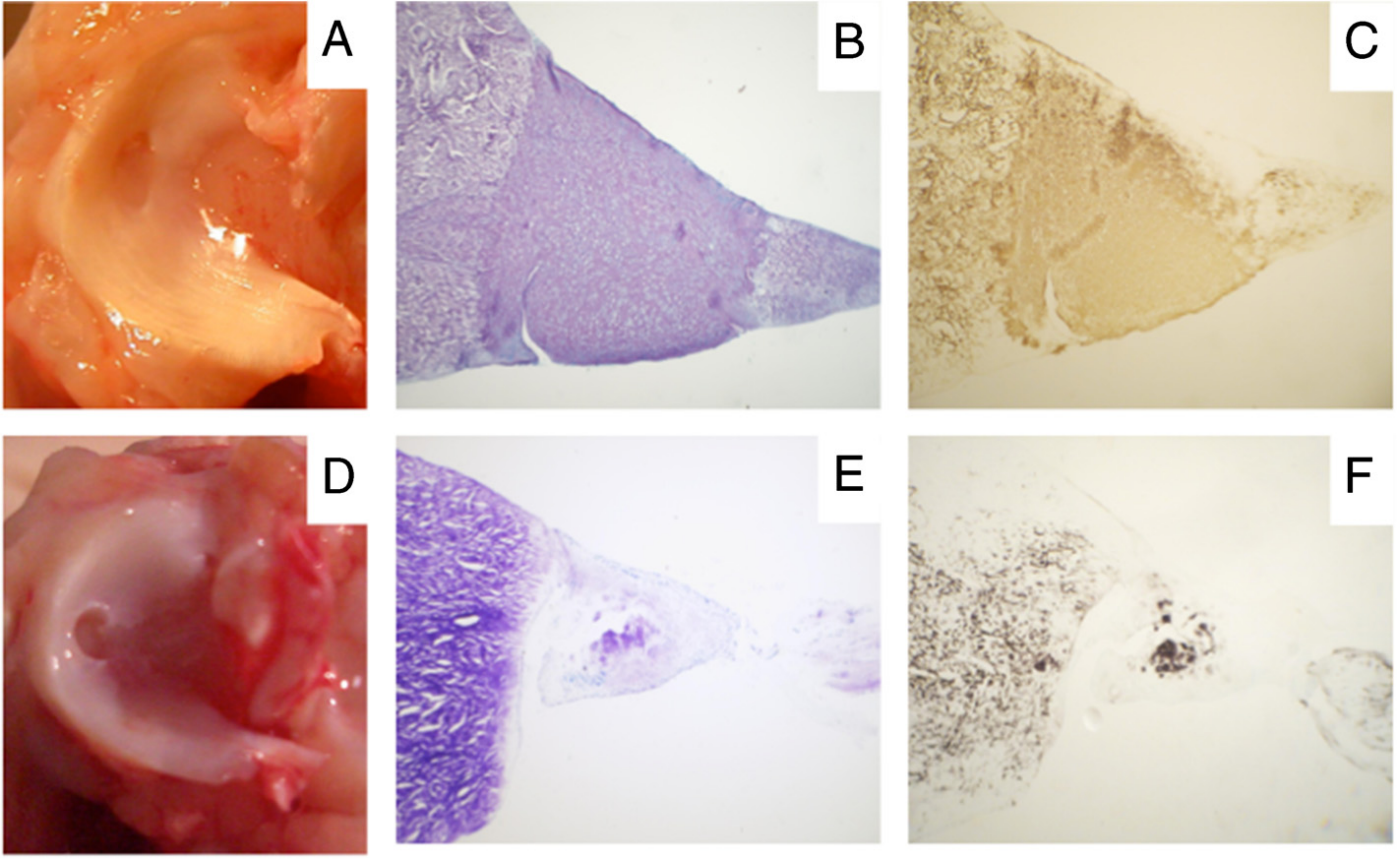
**Treatment of meniscal defects (rabbit) in the avascular zone with a mesenchymal stem cell matrix composite resulted in defect filling with a differentiated meniscus like tissue compared to implantation of a cell free scaffold: Representative macroscopic views, histological (toluidine blue) and immunohistochemical (collagen type II) slides of menisci and the meniscal repair tissue 3 months after treatment of circular meniscal punch defects in the avascular zone; A, B, C: treatment with hyaluronan-collagen composite matrices loaded with mesenchymal stem cells; D, E, F: treatment with a cell-free composite matrix (control).**

Ishimura et al. (1991) showed a faster and improved healing of avascular meniscal defects in a rabbit model by using bone marrow fibrin clot constructs compared to fibrin clot alone (Ishimura et al. 1991). They postulated that the benefit of this treatment is due to the pluripotential stem cells in the bone marrow. In a scaffold-free engineered meniscal tissue Aufderheide and Athanasiou (2007) found a high tensile modulus and improved mechanical properties by a high density co-culture of fibrochondrocytes and mesenchymal stem cells in ring shaped moulds (Aufderheide and Athanasiou 2007). Despite these reults, for in vivo application of mesenchymal stem cells, scaffolds seem to be a useful tool to ensure a lasting effect of the cells directly at the defect site. Yamasaki et al. (2008) using cell-seeded rat decellularized meniscus scaffolds, concluded that cell seeded constructs are more effective than scaffolds alone (Yamasaki et al. 2008). Multiple natural and synthetic scaffolds have been used to deliver cells to the meniscal injury (Sweigart and Athanasiou 2001; Buma et al. 2004; Scotti et al. 2013). A hyluronan collagen composite matrix were found to be suitable for chondrogenic differentiation of mesenchymal stem cells (Angele et al. 2009). Treatment of meniscal full size defects with this scaffold seeded with autologous mesenchymal stem cells after resection of the pars intermedia of the medial meniscus in a rabbit model resulted in a complete defect filling after 3 months in vivo. Only treatment with mesenchymal stem cells was able repair this critical size meniscal defects with stable differentiated meniscus like tissue compared to untreated defects or the treatment with a cellfree hyaluronan collagen scaffold (Angele et al. 2008). Similar results were detected for treatment of isolated avascular meniscal punch defects in the pars intermedia of the lateral meniscus in a rabbit model (Zellner et al. 2010). After 3 months in vivo meniscal defects were filled with differentiated repair tissue after treatment with a hyaluronan collagen composite matrix seeded with mesenchymal stem cells (Figure 4). Interestingly precultured stem cell matrix constructs showed a reduced integration in the native meniscus while non precultured stem cell matrix constructs resulted in a completely integrated repair tissue indicating that also the grade of differentiation of the stem cell matrix construct seems to be an important factor for successful treatment of meniscus with stem cell based Tissue Engineering. In the same model growth factors applied by autologous PRP failed to repair the avascular meniscal punch defect. In summary, mesenchymal stem cells seem to play an essential role for treatment of meniscal defects.

### Ethical approval

The experimental studies were performed with permission of the Ethical comitee at the University Hospital Regensburg.

## Conclusions

Tissue Engineering of meniscus with mesenchymal based cells seems to be a promising approach to treat meniscal tears and defects in order to restore as much native meniscal tissue as possible. In preclinical trials, treatment of meniscus tears only with suture or meniscus defects with cell-free biomaterials, which both represent the actual clinical practice, fail to show comparable results to mesenchymal cell based approaches.

Despite the fact, that meniscus regeneration seems to be feasible by growth factors and mesenchymal stem cells, none of the cell-based strategies has entered clinical practice to date. The implementation of cell-based strategies is mainly limited by regulatory burdens and by the necessity to expand cells prior to transplantation resulting in high treatment costs.

## Consent

Written informed consent was obtained from the patient’s for the publication of this report and any accompanying images.
